# Association of Facial Wrinkles With Different Facial Forms in the Population of Maharashtra: A Prospective Observational Study

**DOI:** 10.7759/cureus.47692

**Published:** 2023-10-25

**Authors:** Sharanbasappa Japatti, Jitendra Kumar, Arif F Merchant, Georgina D Dhalwale, Priyanka Taneja, Rinnu A Mathew

**Affiliations:** 1 Department of Oral and Maxillofacial Surgery, Jawahar Medical Foundation's (JMF's) Annasaheb Chudaman Patil Memorial Medical (ACPM) Dental College, Dhule, IND; 2 Department of Dentistry, All India Institute of Medical Sciences, Bhopal, IND; 3 Department of Periodontics and Oral Implantology, Jawahar Medical Foundation's (JMF's) Annasaheb Chudaman Patil Memorial Medical (ACPM) Dental College, Dhule, IND

**Keywords:** chi-square test, cross sectional studies, photographic analysis, facial profile, facial wrinkles

## Abstract

Introduction: Wrinkles commonly manifest in various areas of the face as individuals age. This study aimed to assess the association between facial wrinkles and different facial forms.

Materials and methods: An observational, prospective study was conducted on the facial photographs of 400 subjects aged 40-60 years, which were divided into four groups of 100 subjects each: Group 1, square facial form; Group 2, ovoid facial form; Group 3, square tapered facial form; and Group 4, tapered facial form. All groups had almost equal distributions of males and females. Six types of facial wrinkles were studied, namely, forehead, glabellar, canthal, nasolabial, wrinkles at the corner of the mouth, and perioral wrinkles. Analysis of variance (ANOVA) was used for intergroup comparison, and an independent Student's t-test was used to assess gender differences in facial wrinkles.

Results: Significant gender differences were observed for forehead wrinkles in Groups 1 and 3, canthal wrinkles in Groups 1 and 2, and right perioral wrinkles in Group 1 (p<0.05). There were non-significant gender differences between right and left-side facial wrinkles (p>0.05). Significant differences between the groups were observed for all facial wrinkles between the right and left sides of the face (p<0.05). There was a significant difference between the groups for the presence of glabellar, corner of the mouth, and perioral wrinkles, with the presence of higher wrinkles in ovoid and tapered facial forms compared to square and square tapered facial forms (p<0.05).

Conclusions: Females had more facial wrinkles than males, predominantly in the forehead region. The least prominent wrinkles were observed in the perioral region of the face. Glabellar, corner of the mouth, and perioral wrinkles were predominantly observed in ovoid and tapered facial forms.

## Introduction

Facial wrinkles are a distinctive attribute of the face that manifests as an individual’s age feature. The occurrence of facial wrinkles depends on the characteristics of the skin and muscle contractions. The formation of wrinkles is also influenced by environmental factors such as sun exposure, air pollution, stress, and smoking [1]. Consequently, the identification of wrinkles plays a crucial role in applications reliant on alterations in the facial skin, such as the estimation of facial age [1]. Age-related alterations in the integumentary system elicit both medical and aesthetic apprehension because of the heightened vulnerability of the skin, the gradual reduction in the thickness of the outermost layer of the skin, the protracted regeneration of wounded tissue, and the escalated occurrence of cutaneous malignancies [2]. Intrinsic factors, namely natural alterations such as the decline in elasticity, tone, and volume of muscles and the remodeling of facial bones, and extrinsic factors, specifically lifestyle modifications such as dietary choices, drug consumption, smoking, and photoaging, can succinctly delineate the factors that affect the aging process [3].

Facial aging can result in detrimental psychological, emotional, and societal consequences from the modification of self-perception and the perception of individuals by others [1]. Wrinkle lines often manifest as signs of aging, specifically forehead and glabellar lines, nasolabial folds, radial lip lines, marionette lines, and lines in the corners of the mouth [4]. It is still not fully understood why there is a discrepancy in the extent of skin wrinkling between males and females, particularly in certain areas. Previous studies have outlined notable variations in perioral wrinkling based on gender and observed that females exhibited more pronounced rhytides than males [5].

It has been noted that several types exist within the realm of facial wrinkles, each with its own unique attributes. These attributes have led to a classification system based on various factors, such as their specific location on the face, the pattern they form, and the depth of penetration. However, despite extensive exploration of this topic, none have garnered as much attention or concern from a cosmetic standpoint as the lines and grooves that arise from facial expressions. The complexity of discussing wrinkles, furrows, and folds stems from the lack of a widely accepted classification system or terminology rooted in anatomical, dimensional, or etiologic criteria. Therefore, comprehensively understanding and categorizing these facial features is challenging.

Because our faces have a unique identity, which means that every individual has their own distinct facial features and characteristics that distinguish them from others, it is important to understand the variability and diversity in the various patterns of wrinkles that can be observed on different faces. Therefore, it is strongly believed that there is a clear and significant correlation between the appearance of different types of wrinkles, which can manifest in various forms, and the specific facial structures and compositions of individuals. Facial forms are classified as square, tapering, square tapering, and ovoid [6].

None of the studies conducted thus far have examined the diverse array of facial wrinkles that manifest in various facial forms. Acquiring a more concise and comprehensive understanding of the manifestation of various patterns of facial wrinkles across different types of facial forms would undeniably mark a significant advancement in the realm of cosmetics while simultaneously serving as a valuable resource for dermatologists, plastic surgeons, and aesthetic surgeons, enabling them to narrow down and refine their treatment approaches. Therefore, the primary objective of this study was to assess and evaluate the distinctive patterns of facial wrinkles that emerge in a diverse range of facial forms. The secondary objectives were to study the pattern of wrinkles and disparities between the right and left sides of the face and to assess potential gender differences in the pattern of these wrinkles.

## Materials and methods

An observational, cross-sectional, single-center prospective study was conducted on facial photographs of patients who attended outpatient clinics in the department of oral and maxillofacial surgery of Annasaheb Chudaman Patil Memorial Medical (ACPM) Dental College, Dhule, India. A convenient sampling method was adopted for the study. Ethical committee approval was obtained from the Institutional Ethical Committee (IEC) before starting the study (approval number: EC/NEW/INST/2022/2959/067). Written informed consent was obtained from all participants.

Sample size estimation

The sample size was calculated using G*Power statistical software ver. 3.1 (Heinrich-Heine-Universität Düsseldorf, Düsseldorf, Germany), with a type I error of 0.05 and a power of 90%. The sample size was calculated to be 360. Considering the 10% sample attrition rate, this study was conducted on 400 subjects.

Participants’ selection and eligibility criteria

After careful screening of 850 subjects, 400 were recruited for the study based on the eligibility criteria of the study. Subjects aged 40-60 years, irrespective of sex, who provided consent for participation were included in the study. Subjects with a history of cosmetic surgery performed in the past six months, such as laser, chemical peeling, botulinum toxin, injectable fillers, and facelift; subjects with systemic disease affecting the skin; pregnant or lactating females; subjects who regularly perform face tightening exercises; subjects with a history of burns or trauma affecting the face; and subjects taking medications that can affect the skin, were excluded from the study.

Four hundred subjects were divided randomly into four groups depending on their facial forms, as follows: Group 1: 100 subjects with square facial form (52 males, 48 females with a mean age of 54.14±2.1 years), Group 2: 100 subjects with ovoid facial form (50 males, 50 females with a mean age of 53.23±1.8 years), Group 3: 100 subjects with square tapered facial form (51 males, 49 females with a mean age of 55.21±1.6 years), and Group 4: 100 subjects with tapered facial form (49 males, 51 females with a mean age of 54.07±2.1 years).

Method for obtaining facial photographs

**Figure 1:**
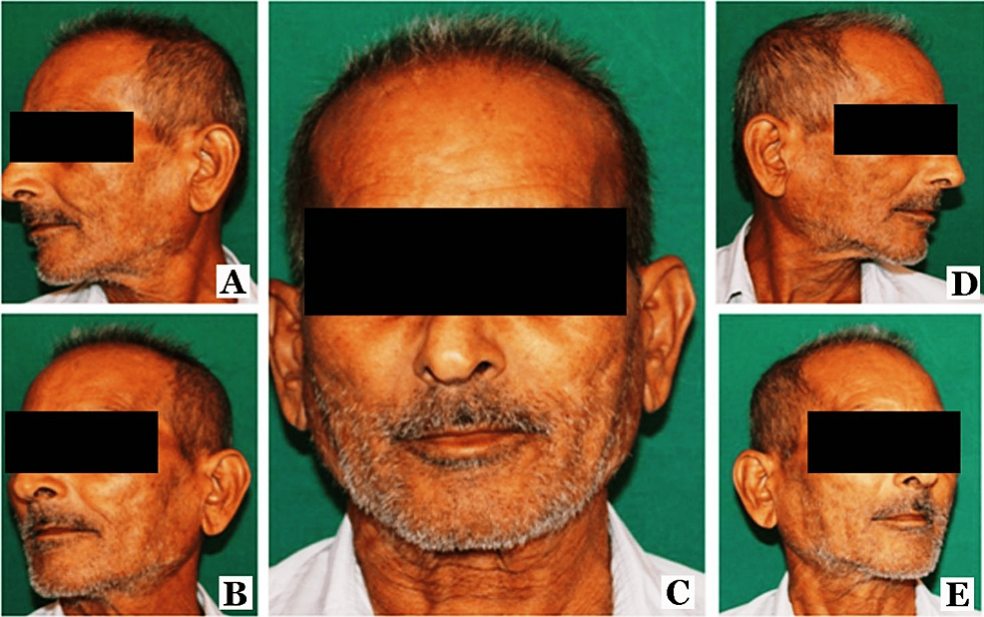
Various photographic views of the participant (A) left profile; (B) left oblique at 45 degrees; (C) frontal; (D) right oblique at 45 degrees; (E) eight profile

Participants were asked to wash their faces with a mild face wash. They were allowed to sit comfortably for 20 minutes in a room with a temperature of 25±0.5°C, and 30±5% relative humidity before starting the procedure. Each subject was photographed by a single trained operator using standard photographic equipment (Canon EOS 1500D digital SLR camera with 24.1-megapixel APS-C CMOS sensor, Canon Zoom Lens EF-S 18-55 mm f/3.5-5.6 IS II, Simpex FC315 Studio Flash Trigger, and Simpex-Pro 300D digital studio camera light photographic equipment) under standard photographic conditions. The subjects were photographed in full face frontal, right and left oblique at 45 degrees, and right and left side profile views with standardized image magnification, appropriate lighting technique, and a standardized camera angle and distance. The various views are presented in Figure 1.

Only static images of the subjects were included in the study, and subjects with various dynamic movements of the facial muscles were advised to reveal a static, normal face with no expressions while taking the photographs.

Evaluation of photographs for facial forms

**Figure 2:**
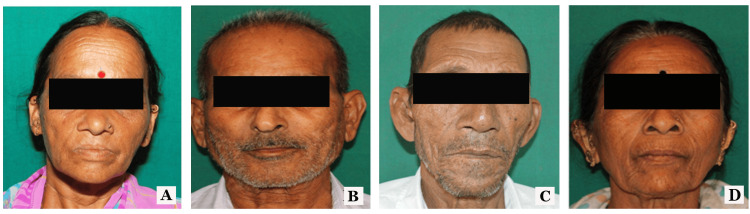
Various facial profiles of the participants (A) ovoid; (B) square; (C) tapered; (D) square tapered

The geometrical method by Ashok et al. was used to categorize patients into four facial forms: square, ovoid, square-tapered, and tapered [6]. The various facial forms are shown in Figure 2.

Evaluation of photographs for facial wrinkles

The facial wrinkles were evaluated at different sites in the subjects: forehead, right and left glabellar, outer canthus, nasolabial, corner of the mouth, and perioral regions, using grades 0-5 of the wrinkle assessment scale by Lemperle et al. in 2001 [4], as shown in Table 1.

**Table 1:** Wrinkle assessment scale by Lemperle et al. [4]

Grade	Score	Sites of facial wrinkles
No visible wrinkles	0	Forehead, glabella, nasolabial, canthal corner of the mouth, perioral
Just perceptible wrinkles	1	
Shallow wrinkles	2	
Moderately deep wrinkles	3	
Deep wrinkles with well-defined edges	4	
Very deep wrinkles, redundant folds	5	

**Figure 3:**
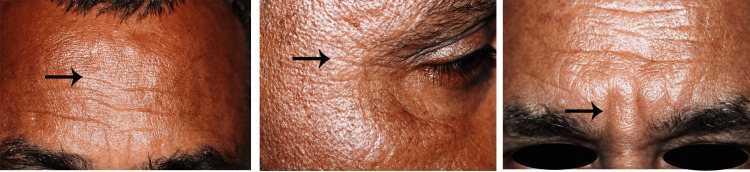
Reference areas marked by an arrowhead for assessment and scoring of wrinkle depth (A) forehead, (B) canthal, and (C) glabellar

**Figure 4:**
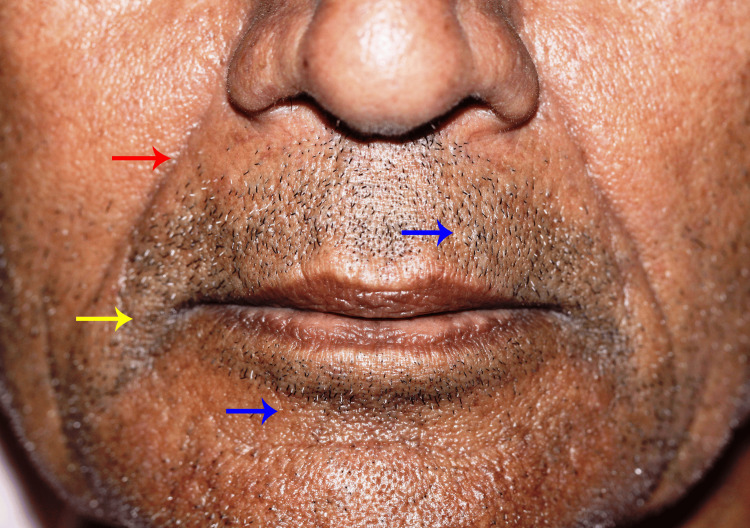
Reference areas for assessment and scoring of wrinkle depth are the nasolabial fold (red arrow), the corner of the mouth (yellow arrow), and the perioral (blue arrows).

The facial wrinkles were evaluated at different facial sites, as shown in Figures 3-4.

Various anatomical landmarks that were already observable on an individual’s face were used to evaluate facial wrinkles. These cardinal points of reference are invariable, and the evaluation of the diverse wrinkles that manifest in specific areas is consistently conducted at identical sites using these identical anatomical benchmarks, as given by Lemperle et al. in 2001 [4]. All photographs were scored by looking at them only once, and they were scored at the first sight of the examination to avoid any discrepancy or change in scoring.

Reliability of assessment and blinding

Two oral surgeons evaluated the patients for their facial forms, and two evaluators (oral surgeons with more than 10 years of experience), who were blinded to the facial forms and groups, independently scored all the photographs using the wrinkle assessment scale. After two weeks, the scoring was repeated on 100 randomly selected photographs by the same evaluators who were blinded to their previous scores. Inter-rater and intra-rater reliabilities were assessed using the intra-class correlation coefficient (ICC).

Statistical analysis

The intra-class correlation coefficient showed a high intra-rater reliability of 92%-96% and a high inter-rater reliability of 88%-92%. The mean facial wrinkling score was determined by averaging the grades of both evaluators. Data entry and analysis were performed using Microsoft Excel (Microsoft Corp., Redmond, WA) and IBM SPSS software version 22 (IBM Corp., Armonk, NY) for statistical analysis. The Shapiro-Wilk test was used to check the normality of the data, and the data were found to be normally distributed. Descriptive statistics were applied for all wrinkle patterns in all facial forms in both sexes. The gender differences in the facial wrinkles on the right and left sides of the face and in different groups were assessed by an independent student t-test. Intergroup comparison was performed by analysis of variance (ANOVA), followed by post-hoc analysis by Tukey's test. Differences were considered statistically significant at p<0.05.

## Results

Sex-related differences in the scores for facial wrinkles

The gender differences in the mean score values of facial wrinkles in four study groups are shown in Table 2.

**Table 2:** Gender differences in the mean score values of facial wrinkles in four study groups using an independent Student's t-test *p value<0.05: significant; **p value<0.001: highly significant; SD: standard deviation

Wrinkle site	Group 1 (Mean±SD)			Group 2 (Mean±SD)			Group 3 (Mean±SD)			Group 4 (Mean±SD)		
	Female	Male	p-value	Female	Male	p-value	Female	Male	p-value	Female	Male	p-value
Forehead	2.93±1.12	2.85±1.76	0.02*	2.79±1.34	2.81±1.65	0.06	2.7±1.65	2.31±1.45	0.04*	2.65±1.16	2.81±1.21	0.36
Right glabellar	1.73±1.09	1.39±1.27	0.28	1.65±1.09	1.6±1.54	0.45	1.1±1.45	1.08±1.56	0.81	1.09±1.08	1.08±1.13	0.89
Left and center glabellar	1.86±1.32	1.56±1.21	0.16	1.66±1.01	1.6±1.34	0.71	1.4±1.21	1.35±1.89	0.79	1.43±1.78	1.36±1.10	0.68
Right canthal	2.29±1.07	1.91±1.32	0.03*	2.19±1.65	1.98±1.21	0.04*	2.13±1.56	1.85±1.21	0.50	2.04±1.12	2.26±1.21	0.28
Left canthal	2.27±1.18	1.91±1.32	0.04*	2.19±1.67	1.98±1.71	0.04*	2.13±1.12	1.85±1.34	0.50	2.04±1.23	2.26±1.45	0.28
Right nasolabial	2.78±1.32	2.49±1.56	0.66	2.59±1.32	2.42±1.34	0.19	2.83±1.34	2.38±1.34	0.05	2.53±1.76	2.73±1.12	0.93
Left nasolabial	2.78±1.23	2.42±1.54	0.51	2.59±1.45	2.48±1.67	0.65	2.83±1.78	2.38±1.56	0.05	2.53±1.89	2.69±1.23	0.95
Right mouth corner	1.01±0.98	0.63±0.43	0.19	0.45±0.18	0.39±0.17	0.47	0.96±1.32	0.7±0.34	0.43	0.97±1.23	0.93±0.56	0.80
Left mouth corner	1.06±1.12	0.6±0.54	0.12	0.45±0.21	0.35±0.21	0.32	0.93±1.12	0.67±0.45	0.41	0.92±0.98	0.81±0.32	0.99
Right perioral	0.57±0.34	0.21±0.32	0.02*	0.06±0.03	0.08±0.05	0.50	0.6±0.34	0.31±0.12	0.34	0.75±0.23	0.71±0.67	0.35
Left perioral	0.57±0.78	0.21±0.18	0.09	0.06±0.02	0.08±0.02	0.50	0.56±0.23	0.29±0.07	0.36	0.7±0.45	0.59±0.34	0.65

In both genders, maximum scores were observed for forehead wrinkles in all facial forms, and minimum scores were observed in the area of perioral wrinkles.

Forehead wrinkle scores were highest for females in Group 1. There were significant differences in mean forehead scores in Group 1, and Group 3 (p<0.05). In general, females displayed more forehead wrinkles than males in all facial forms, except for the tapered form. Females presented with more glabellar, nasolabial, and mouth corner wrinkles, compared with males in all facial forms, although the difference was not statistically significant (p>0.05). Females showed higher canthal wrinkles than males, which were statistically significant for Group 1 and Group 2 (p<0.05). The presence of perioral wrinkles was lowest compared with other wrinkles in all the facial forms in both sexes. Females had significantly more perioral wrinkles on the right side, compared to males in Group 1(p<0.05).

Side-related differences in facial wrinkles

On comparing the mean wrinkle scores of the right and left sides in males and females, non-statistically significant differences were found for all the facial wrinkles (p>0.05), as shown in Table 3.

**Table 3:** Comparative analysis of the right and left wrinkle sites in relation to gender using an independent Student's t-test

Wrinkle site	Gender	Mean	Standard deviation	Variance	p-value
Right glabellar	Female	1.495	1.235	1.526	0.06
	Male	1.277	1.262	1.592	
Left and center glabellar	Female	1.644	1.196	1.43	0.12
	Male	1.466	1.202	1.444	
Right canthal	Female	2.185	1.13	1.277	0.061
	Male	1.973	1.118	1.25	
Left canthal	Female	2.181	1.133	1.284	0.055
	Male	1.973	1.118	1.25	
Right nasolabial	Female	2.671	1.078	1.161	0.08
	Male	2.489	1.183	1.399	
Left nasolabial	Female	2.671	1.078	1.161	0.06
	Male	2.473	1.173	1.376	
Right mouth corner	Female	0.782	0.966	0.934	0.15
	Male	0.663	0.874	0.764	
Left mouth corner	Female	0.782	0.966	0.934	0.06
	Male	0.625	0.85	0.722	
Right perioral	Female	0.412	0.864	0.746	0.17
	Male	0.314	0.721	0.521	
Left perioral	Female	0.398	0.878	0.771	0.11
	Male	0.284	0.68	0.463	

Therefore, the mean wrinkle score values of males and females on the right and left sides were taken together for further analysis. Statistically significant differences were observed for all facial wrinkles in all facial forms between the right and left sides of the face (p<0.05), as shown in Table 4.

**Table 4:** Comparison of mean wrinkle scores between different groups in relation to the right and left sides of the face using an independent Student's t-test *p value<0.05: significant; **p value<0.001: highly significant; SD: standard deviation

Wrinkle site	Group 1 (Mean±SD)			Group 2 (Mean±SD)			Group 3 (Mean±SD)			Group 4 (Mean±SD)		
	Right	Left	p-value	Right	Left	p-value	Right	Left	p-value	Right	Left	p-value
Glabellar	1.55±1.32	1.24±1.22	.003*	1.63±1.13	1.72±1.04	.033*	1.08±1.27	1.36±1.17	.001**	1.68±1.17	1.42±1.26	.001**
Canthal	2.32±1.15	2.61±1.08	.012*	2.02±1.14	2.74±1.52	.001**	1.92±1.05	2.02±0.85	.001**	2.16±1.14	1.19±1.04	.001**
Nasolabial	2.62±1.16	2.51±1.21	.042*	2.52±1.18	2.01±1.14	.015*	2.54±1.05	1.92±1.05	.001**	2.64±1.14	1.82±1.09	.001**
Mouth corner	0.71±0.12	0.67±0.22	.001**	0.91±0.14	0.98±1.12	.021*	0.87±0.14	0.73±0.12	.014*	0.95±0.13	0.86±0.12	.031*
Perioral	0.37±0.14	0.29±0.78	.001**	0.87±0.25	0.95±0.19	.013*	0.39±0.77	0.26±0.18	.001**	0.93±0.14	0.64±1.05	.021*

There were more glabellar wrinkles on the left side of the face than on the right side in Groups 2 and 3. Maximum wrinkles were observed in Groups 1 and 4. The canthal wrinkles were highest in Group 1 and lowest in Group 3. The higher canthal wrinkles were present on the left side of the face, compared to the right side, in all the groups except Group 4. Nasolabial wrinkles were the most predominant wrinkles in all facial forms and were highest in Group 4 on the right side of the face, followed by Group 1. These wrinkles were higher on the right side of the face than on the left side in all facial forms. The wrinkles at the corner of the mouth were higher on the right side of the face in all facial forms, except for Group 2. Groups 2 and 4 displayed higher wrinkles compared to Groups 1 and 3. The perioral wrinkles observed on the face were found to be the least prominent in comparison to the other wrinkles, particularly in Group 1. These wrinkles were predominantly seen more on the right side of the face compared to the left side in all the groups except Group 2.

Association between wrinkles and facial forms

On assessing the association of facial wrinkles at different anatomical sites with different facial forms in both genders, it was noticed that both males and females showed higher mouth corners and perioral wrinkles in ovoid and tapered facial forms compared to square and square tapered forms. Females showed higher glabellar wrinkles than males, more in ovoid and tapered facial forms, as shown in Table 5.

**Table 5:** Intergroup comparison of mean wrinkle scores between groups and wrinkle sites in relation to gender using the ANOVA test *p value<0.05: significant; **p value<0.001: highly significant; SD: standard deviation

Wrinkle site	Gender	Group 1 (Mean±SD)	Group 2 (Mean±SD)	Group 3 (Mean±SD)	Group 4 (Mean±SD)	p-value
Forehead	Male	2.93±1.38	2.79±1.26	2.70±1.05	2.65±1.54	0.06
	Female	2.85±1.34	2.82±1.39	2.31±1.28	2.81±1.36	0.741
Glabellar	Male	1.80±1.37	1.66±1.06	1.25±1.18	1.26±1.06	0.16
	Female	1.47±1.10	1.60±1.21	1.21±1.19	1.22±1.21	0.05
Canthal	Male	2.28±1.26	2.19±1.09	2.13±1.04	2.04±1.09	0.20
	Female	1.91±1.03	1.98±1.22	1.85±1.05	2.26±1.18	0.76
Nasolabial	Male	2.78±1.15	2.59±1.07	2.83±0.79	2.53±1.14	0.46
	Female	2.45±1.19	2.45±1.27	2.38±1.10	2.71±1.13	0.48
Mouth corner	Male	1.04±1.01	1.45±0.66	0.95±1.00	1.25±1.17	0.023*
	Female	0.42±0.80	0.68±0.70	0.52±0.76	0.87±1.13	0.001**
Perioral	Male	0.57±0.89	0.86±0.23	0.58±1.00	0.73±1.24	0.001**
	Female	0.21±0.55	0.38±0.28	0.30±0.66	0.65±1.05	0.001**

Glabellar, mouth corner, and perioral wrinkles on both sides of the face also showed a higher predilection for ovoid and tapered facial forms (Tables 6-8).

**Table 6:** Intergroup comparison of mean facial wrinkle scores in four study groups according to the right and left sides using the ANOVA test *p value<0.05: significant; SD: standard deviation

Wrinkle site	Side	Group 1 (Mean±SD)	Group 2 (Mean±SD)	Group 3 (Mean±SD)	Group 4 (Mean±SD)	p-value
Glabellar	Right	1.55±1.32	1.63±1.13	1.08±1.27	1.68±1.17	0.01*
	Left	1.24±1.22	1.72±1.04	1.36±1.17	1.42±1.26	0.04*
Canthal	Right	2.32±1.15	2.02±1.14	1.92±1.05	2.16±1.14	0.41
	Left	2.61±1.08	2.74±1.52	2.02±0.85	1.19±1.04	0.42
Nasolabial	Right	2.62±1.16	2.52±1.18	2.54±1.05	2.64±1.14	0.71
	Left	2.51±1.21	2.01±1.14	1.92±1.05	1.82±1.09	0.84
Mouth corner	Right	0.71±0.12	0.91±0.14	0.87±0.14	0.95±0.13	0.01*
	Left	0.67±0.22	0.98±1.12	0.73±0.12	0.86±0.12	0.013*
Perioral	Right	0.37±0.14	0.87±0.25	0.39±0.77	0.93±0.14	0.02*
	Left	0.29±0.78	0.95±0.19	0.26±0.18	0.64±1.05	0.03*

**Table 7:** Intergroup comparative analysis of wrinkle score with facial profile using the ANOVA test *p value<0.05: significant; **p value<0.001: highly significant; SD: standard deviation

Wrinkle site	Group 1 (Mean±SD)	Group 2 (Mean±SD)	Group 3 (Mean±SD)	Group 4 (Mean±SD)	F-value	p-value
Forehead	2.29±1.23	2.80±1.44	2.41±1.36	2.74±1.31	0.813	0.31
Glabellar	1.39±1.18	1.68±1.14	1.22±1.24	1.54±1.12	4.579	0.004*
Canthal	2.26±1.05	2.38±1.14	1.97±1.15	2.16±1.11	0.942	0.42
Nasolabial	2.51±1.04	2.63±1.13	2.52±1.18	2.63±1.15	0.33	0.804
Mouth corner	0.69±0.83	0.95±1.14	0.75±0.92	0.91±0.67	7.311	.01*
Perioral	0.27±0.46	0.91±0.22	0.32±0.45	0.78±0.56	12.66	.001**

**Table 8:** Post-hoc analysis of wrinkle scores in different groups using Tukey's test (only significant comparison values are displayed in the table) *p value<0.05: significant; **p value<0.001: highly significant

Wrinkle site	Groups		p-value
Glabellar	Group 3	Group 2	0.02*
Mouth corner	Group 3	Group 2	0.01*
	Group 1	Group 2	0.001**
	Group 4	Group 2	0.001**
Perioral	Group 3	Group 2	0.007*
	Group 1	Group 2	0.004*
	Group 4	Group 2	0.001**
	Group 3	Group 4	0.01*
	Group 1	Group 4	0.01*

## Discussion

Wrinkles manifest due to changes at the dermal-epidermal junction, enduring a decline in papillae and degeneration of elastic and collagen fibers. This degenerative process starts as early as 30 years of age and progressively intensifies over time, irrespective of diligent maintenance and safeguarding measures [7]. Facial aging primarily arises from the phenomenon of bone displacement and development as well as the consequential distortions in the skin that manifest as wrinkles and a decline in muscular vigor. Bone growth predominantly occurs during childhood, whereas the most significant transformations associated with aging during adulthood are predominantly characterized by alterations in texture [8].

Age-related changes are predominantly seen after the age of 40 years, and therefore, this study was conducted on individuals aged 40-60 years. Before reaching the milestone of 40 years, the manifestation of fine lines becomes more conspicuous compared to wrinkles. Fine lines serve as the precursor to wrinkles and are characterized by minuscule creases on the surface of the dermis. In contrast, wrinkles are situated at a greater depth within the skin. It was revealed from the study that females had predominantly more wrinkles than males. The most wrinkles were noticed in the forehead region, and the least in the perioral area. Our findings were in accordance with the previous study reported by Brown et al. [9]. Collagen 1 and collagen 3 constitute the primary constituents of the reticular and papillary dermis and are synthesized by dermal fibroblasts, which represent the principal cellular components of the dermis. Females showed higher expression of collagen 3 in the perioral area, which could be a reason for less wrinkling in this area [9]. The occurrence of wrinkles around the mouth is also reduced in males, possibly because of the photoprotective properties of male terminal hairs and the heightened rate of cell turnover in the hair follicles responsible for their production [5]. Increased wrinkles in the forehead region may be due to solar elastosis caused by ultraviolet (UV) exposure to the sun [10]. Females show more wrinkles than males, which could be due to the fact that females might also encounter amplified matrix metalloprotein (MMP-1) synthesis, as demonstrated by the escalated cysteine-rich angiogenic inducer 61 (CYR61) manifestation in the facial area, intensifying the decline of collagen 1 and the manifestation of wrinkles [9]. However, Tsukahara et al. found that males had more facial wrinkles than females of all ages. The reason for this disparity could be the small sample size of 22, and in their study, significant differences were seen in younger age groups of less than 40 years [11].

The elasticity of the skin lacks support and consequently forms periorbital wrinkles. This is further accentuated by the reduction in the subcutaneous tissue located beneath the infraorbital region, intensifying the impact of the inherent tension within the orbicularis oculi muscle on the skin that lies above it, resulting in the emergence of wrinkles at the outer corner of the eye [12]. Transverse creases on the forehead arise from the contraction of the frontalis muscle, which occurs in a direction perpendicular to the muscle's movement. On the other hand, the presence of more vertical wrinkles is associated with what is commonly known as "sleep crunch lines." Glabellar furrows and nasal root horizontal lines are predominantly induced by contraction of the corrugator supercilii and procerus muscles, respectively. Perioral wrinkles are mainly caused by changes in the orbicularis oris muscle [1]. The progression of facial asymmetry with age leads to a conspicuous transformation in facial landmarks, the emergence of wrinkles, and the occurrence of sagging [13]. The differences in wrinkles between the right and left sides of the face observed in the present study could be due to the different sleep postures of the subjects. Facial wrinkles are formed as a result of the pressure exerted on the face during sleep, thereby promoting facial skin expansion [14].

Every person has a unique type of facial form, and because there is no particular type of wrinkle pattern that exists in two different individuals, this study was conducted to evaluate whether there is any association between the presence of various types of wrinkles and facial forms. The results of this study indicated that the presence of glabellar and perioral wrinkles was significantly associated with ovoid and tapered facial forms. To date, no study has been conducted on the association between wrinkles in different facial forms. According to Swift et al., facial aging is a multifaceted, interconnected, three-dimensional phenomenon that encompasses alterations in the skeletal framework, soft tissues, and the integumentary system [1]. Although each anatomical stratum experiences its own aging process, there is also a reliance on more superficial structures in the underlying layers. Despite the relatively minor scale of skeletal alterations occurring in the most profound stratum of facial morphology, repercussions frequently exhibit a substantial impact [1].

The ovoid and tapered facial forms are associated with a symphysis characterized by considerable height, limited depth, substantial ratio, minimal angle, decreased height and width of the ramus, reduced depth of the mandible, increased gonial angle, and diminished arc angle of the mandible, in contrast to the square and square tapered forms of the mandible [15]. Ovoid and tapered facial forms are also associated with decreased bigonial and bizygomatic widths, downward rotation of the face relative to the cranial base, and clockwise rotation of the maxillary base with flattening of facial angles compared with square facial forms [16]. It has been observed in previous studies that decreased bigonial widths, flattening of facial angles, and clockwise rotation of the maxilla and mandible are associated with more wrinkles [1].

Ovoid and tapered facial forms are also associated with weaker facial musculature than square facial forms [17]. The facial musculature attaches to the dermal layer, thereby exerting a significant influence on both the support and overall structural stability of the surrounding soft tissue. Consequently, this directly affects the size and shape of the area in question [18]. Therefore, differences in bone and muscle morphology in different facial types affect changes in overlying soft tissues, leading to differences in the appearance of wrinkles in different facial forms.

Limitations of the study

This was a single-center photographic study. The measurement of wrinkles is a complex process that involves various tools, methods and scientifically proven and universally accepted assessment scales. The use of casts, impressions, digital scopes, or any digital software for the assessment of facial wrinkles in the photographs of the study could help in an in-depth understanding of the relationship between facial wrinkles and facial forms. A universally accepted wrinkle assessment scale that includes treatment along with a thorough classification of wrinkles over the face is still not found in the literature. Furthermore, the current investigation was carried out utilizing a convenient sampling technique, potentially compromising the generalizability of the study's results. Additionally, the study did not take into consideration any potential racial or ethnic variations.

Clinical implications of the study

The present study can help professionals who want to treat photoaging target their therapy over proven areas, such as the glabellar, perioral, and corner of the mouth regions, in a minimally invasive manner, which will in turn provide patient satisfaction in a limited period of time. Cosmetic procedures are required more in females and in ovoid and tapered facial forms, mainly in the perioral regions. Further clinical trials in continuation of this study involving various non-invasive procedures for the treatment of the commonly observed wrinkles in this study according to a particular type of face form will definitely bring a change in the treatment modalities in cosmetics.

## Conclusions

Within the limitations of this study, it was concluded that a significant association exists between facial wrinkles and facial forms. Glabellar, mouth corner, and perioral wrinkles were predominantly observed in ovoid and tapered facial forms compared with square and square tapered facial forms. Females had more wrinkles than males, predominantly in the forehead region. The fewest wrinkles were observed in the perioral region of the face. Significant differences were observed in wrinkles between the right and left sides of the face.

## References

[1] Swift A, Liew S, Weinkle S, Garcia JK, Silberberg MB (2021). The facial aging process from the “inside out”. Aesthet Surg J.

[2] Keaney TC (2016). Aging in the male face: intrinsic and extrinsic factors. Dermatol Surg.

[3] Vashi NA, de Castro Maymone MB, Kundu RV (2016). Aging differences in ethnic skin. J Clin Aesthet Dermatol.

[4] Lemperle G, Holmes RE, Cohen SR, Lemperle SM (2001). A classification of facial wrinkles. Plast Reconstr Surg.

[5] Chien AL, Qi J, Cheng N (2016). Perioral wrinkles are associated with female gender, aging, and smoking: Development of a gender-specific photonumeric scale. J Am Acad Dermatol.

[6] Ashok V, Ganapathy D (2019). A geometrical method to classify face forms. J Oral Biol Craniofac Res.

[7] Kaur M, Garg RK, Singla S (2015). Analysis of facial soft tissue changes with aging and their effects on facial morphology: A forensic perspective. Egypt J Foren Sci.

[8] Mendelson B, Wong CH (2012). Changes in the facial skeleton with aging: implications and clinical applications in facial rejuvenation. Aesthetic Plast Surg.

[9] Brown ID, Dillen C, Ly BC, Shyam N, Kang S, Chien AL (2023). Sex-specific differences in oxidative stress markers and collagen expression in perioral skin wrinkling. Exp Dermatol.

[10] Tsukahara K, Tamatsu Y, Sugawara Y, Shimada K (2012). Morphological study of the relationship between solar elastosis and the development of wrinkles on the forehead and lateral canthus. Arch Dermatol.

[11] Tsukahara K, Hotta M, Osanai O, Kawada H, Kitahara T, Takema Y (2013). Gender-dependent differences in degree of facial wrinkles. Skin Res Technol.

[12] Kane MA (2003). Classification of crow's feet patterns among Caucasian women: the key to individualizing treatment. Plast Reconstr Surg.

[13] Ercan I, Ozdemir ST, Etoz A, Sigirli D, Tubbs RS, Loukas M, Guney I (2008). Facial asymmetry in young healthy subjects evaluated by statistical shape analysis. J Anat.

[14] Anson G, Kane MA, Lambros V (2016). Sleep wrinkles: facial aging and facial distortion during sleep. Aesthet Surg J.

[15] Mangla R, Singh N, Dua V, Padmanabhan P, Khanna M (2011). Evaluation of mandibular morphology in different facial types. Contemp Clin Dent.

[16] Knigge RP, McNulty KP, Oh H (2021). Geometric morphometric analysis of growth patterns among facial types. Am J Orthod Dentofacial Orthop.

[17] Agrawal A, Nandini A, Das M (2022). Contribution of masticatory muscle pattern in craniofacial morphology: a systematic review. J Indian Orthod Soc.

[18] Swift A, Remington BK (2019). The mathematics of facial beauty. Injectable Fillers: Facial Shaping and Contouring.

